# Complementing Prostate SBRT VMAT With a Two-Beam Non-Coplanar IMRT Class Solution to Enhance Rectum and Bladder Sparing With Minimum Increase in Treatment Time

**DOI:** 10.3389/fonc.2021.620978

**Published:** 2021-03-19

**Authors:** Abdul Wahab M. Sharfo, Linda Rossi, Maarten L. P. Dirkx, Sebastiaan Breedveld, Shafak Aluwini, Ben J. M. Heijmen

**Affiliations:** ^1^ Department of Radiotherapy, Erasmus MC Cancer Institute, University Medical Center, Rotterdam, Netherlands; ^2^ Department of Radiation Oncology, University Medical Center Groningen, Groningen, Netherlands

**Keywords:** non-coplanar, beam angle optimization, class solution, automated planning, prostate SBRT

## Abstract

**Purpose:**

Enhance rectum and bladder sparing in prostate SBRT with minimum increase in treatment time by complementing dual-arc coplanar VMAT with a two-beam non-coplanar IMRT class solution (CS).

**Methods:**

For twenty patients, an optimizer for automated multi-criterial planning with integrated beam angle optimization (BAO) was used to generate dual-arc VMAT plans, supplemented with five non-coplanar IMRT beams with individually optimized orientations (VMAT+5). In all plan generations, reduction of high rectum dose had the highest priority after obtaining adequate PTV coverage. A CS with two most preferred directions in VMAT+5 and largest rectum dose reductions compared to dual-arc VMAT was then selected to define VMAT+CS. VMAT+CS was compared with automatically generated *i)* dual-arc coplanar VMAT plans (VMAT), *ii)* VMAT+5 plans, and *iii)* IMRT plans with 30 patient-specific non-coplanar beam orientations (30-NCP). Plans were generated for a 4 x 9.5 Gy fractionation scheme. Differences in PTV doses, healthy tissue sparing, and computation and treatment delivery times were quantified.

**Results:**

For equal PTV coverage, VMAT+CS, consisting of dual-arc VMAT supplemented with two fixed, non-coplanar IMRT beams with fixed Gantry/Couch angles of 65°/30° and 295°/-30°, significantly reduced OAR doses and the dose bath, compared to dual-arc VMAT. Mean relative differences in rectum D_mean_, D_1cc_, V_40GyEq_ and V_60GyEq_ were 19.4 ± 10.6%, 4.2 ± 2.7%, 34.9 ± 20.3%, and 39.7 ± 23.2%, respectively (all *p*<0.001). There was no difference in bladder D_1cc_, while bladder D_mean_ reduced by 17.9 ± 11.0% (*p*<0.001). Also, the clinically evaluated urethra D_5%_, D_10%_, and D_50%_ showed small, but statistically significant improvements. All patient V_X_ with X = 2, 5, 10, 20, and 30 Gy were reduced with VMAT+CS, with a maximum relative reduction for V_10Gy_ of 19.0 ± 7.3% (*p*<0.001). Total delivery times with VMAT+CS only increased by 1.9 ± 0.7 min compared to VMAT (9.1 ± 0.7 min). The dosimetric quality of VMAT+CS plans was equivalent to VMAT+5, while optimization times were reduced by a factor of 25 due to avoidance of individualized BAO. Compared to VMAT+CS, the 30-NCP plans were only favorable in terms of dose bath, at the cost of much enhanced optimization and delivery times.

**Conclusions:**

The proposed two-beam non-coplanar class solution to complement coplanar dual-arc VMAT resulted in substantial plan quality improvements for OARs (especially rectum) and reduced irradiated patient volumes with minor increases in treatment delivery times.

## Introduction

Stereotactic body radiation therapy (SBRT) is becoming the standard treatment radiotherapy option for several primary and metastatic tumors (1–8). In prostate SBRT, volumetric modulated arc therapy (VMAT) has been promoted because of its short treatment delivery time (9–11). On the other hand, several studies have shown that use of non-coplanar beam arrangements minimizes doses in the normal tissues compared to coplanar VMAT, at the cost of enhanced treatment delivery times (12–14). To overcome the prolonged treatment times of non-coplanar IMRT beam arrangements, recent work has been focusing on increasing the delivery efficiency by employing non-coplanar arcs instead. This showed promise due to a drastic reduction in the treatment time, while obtaining a high plan quality (15–18). Recently, we proposed a novel treatment approach for liver SBRT, designated VMAT+, complementing VMAT with a few non-coplanar IMRT beams with computer-optimized, patient-specific orientations to enhance plan quality, while keeping delivery time low (19). However, plan optimization times for VMAT+ were long, in the magnitude of 2-3 days, because of the need for individually optimized beam angles (BAO).

In this study, we used our in-house developed algorithm for automated multi-criterial planning with integrated BAO to explore opportunities for enhancing prostate SBRT dose distributions by complementing dual-arc coplanar VMAT with non-coplanar IMRT beams. To keep the total delivery time limited, the investigated maximum number of complementary non-coplanar beams was five (VMAT+5). Based on the selected beam orientations in the VMAT+5 plans, a class solution (CS) for the non-coplanar beams was defined. Final VMAT+CS plans were benchmarked against automatically generated *i)* dual-arc coplanar VMAT plans (VMAT), *ii)*, VMAT+5 plans, and *iii)* 30-beam non-coplanar IMRT plans with computer-optimized beam orientations (30-NCP). Differences in dosimetric plan parameters, computation and treatment delivery times were analyzed. Dose measurements were performed to verify deliverability of generated plans at the treatment unit.

## Materials and Methods

### Patient Data

Planning CT-scans of 20 randomly selected, low-risk prostate SBRT patients were included in this study. In all CT-scans the rectum (outer contour), rectal mucosa (3mm wall), bladder, urethra, femoral heads, scrotum, penis and prostate were delineated. Patients had implanted fiducials for image-guidance. The average planning target volume (PTV) size was 91.2 cc [57.8 – 142.3 cc] (PTV was defined as prostate plus 3 mm isotropic margin). Dose was delivered in 4 fractions of 9.5 Gy (38 Gy total dose), emulating high-dose rate (HDR) brachytherapy with highly heterogeneous dose distributions in the peripheral zone while restricting the urethra dose (20). PTV coverage aim was 95%, with a maximum dose of 62.5 Gy. Plan acceptability was subject to the dosimetric constraints presented in **Table 1**. While always staying withing imposed hard constraints (**Table 1**), the primary planning aim was to obtain adequate target coverage, followed by a reduction of high rectum doses and other OAR and patient dose reductions. In line with the ALARA principle, the aim was to always maximally reduce healthy tissue doses.

**Table 1:** Clinically applied dose constraints for prostate SBRT.

Structure	Parameter	Tolerance Limit
**PTV**	D_max_	62.5 Gy
**Rectum**	D_max_	38 Gy
	D_1cc_	32.3 Gy
**Rectum mucosa**	D_max_	28.5 Gy
**Bladder**	D_max_	41.8 Gy
	D_1cc_	38 Gy
**Urethra**	D_5%_	45.5 Gy
	D_10%_	42 Gy
	D_50%_	40 Gy
**Femoral heads**	D_max_	24 Gy
**Penis/Scrotum**	D_max_	1.5 Gy

### Automated Plan Generation

For each patient, the Erasmus-iCycle multi-criterial optimizer was used to automatically generate one treatment plan per investigated technique (VMAT, VMAT+CS, VMAT+5, 30-NCP) that is both Pareto-optimal and clinically favorable (21). For practical and legal reasons, Erasmus-iCycle plans are not directly used clinically. Instead, the Erasmus-iCycle plan is automatically converted into a clinically deliverable plan by the Monaco treatment planning system (TPS) (Elekta AB, Stockholm, Sweden) (22). For this purpose, a patient-specific Monaco template is automatically made based on the Erasmus-iCycle dose distribution. In case plan generation includes BAO, the optimal angles are established with Erasmus-iCycle, and are then used as fixed angles in the subsequent Monaco plan generation. Many studies have demonstrated superiority of these automatically generated plans compared to manually generated plans (14, 23–26).

**Figure 1:**
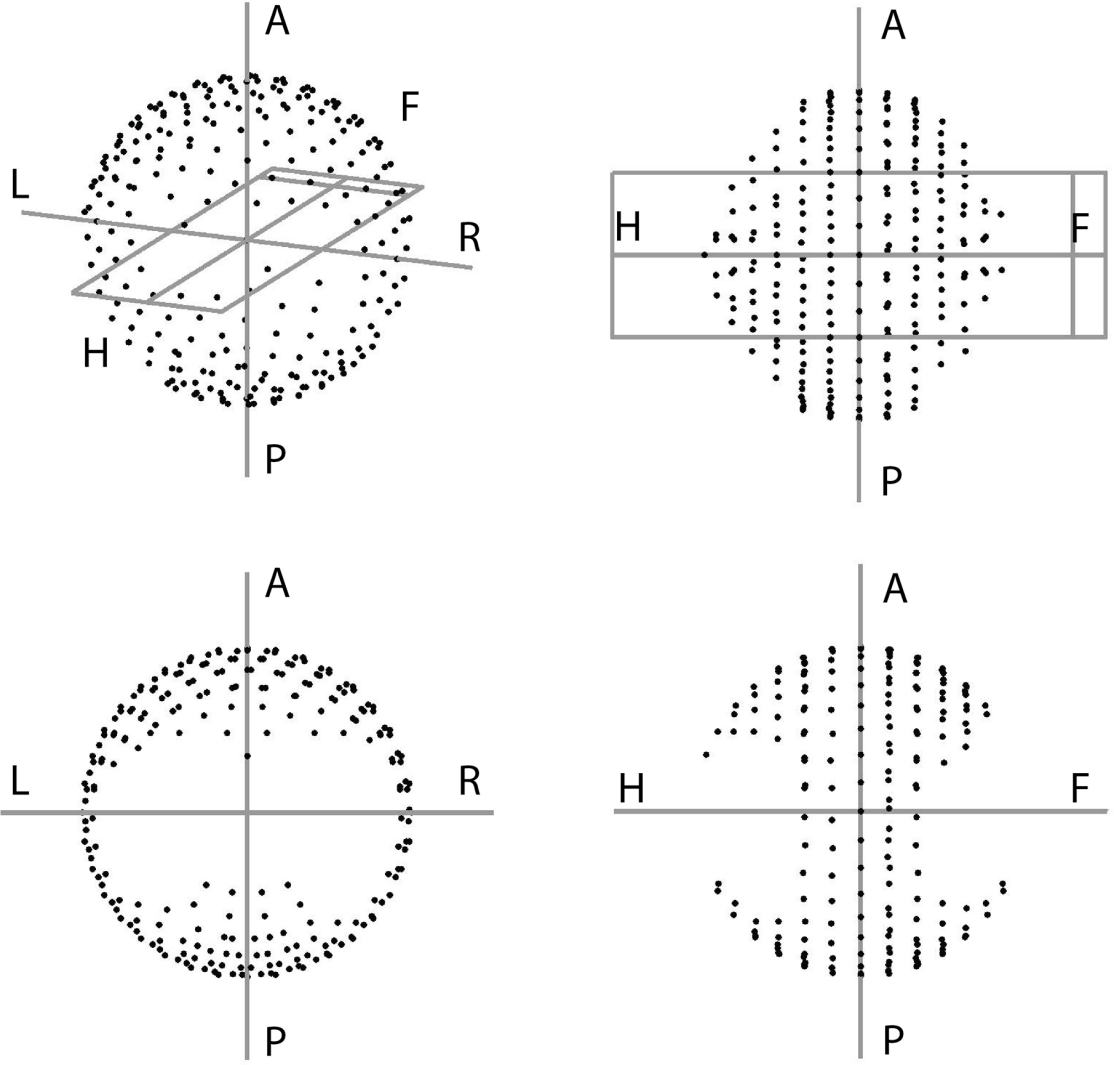
The full beam-angle search space for linac-based prostate SBRT planning as defined on our treatment unit using head and knees supporting devices.

Multi-criterial plan generation with Erasmus-iCycle is based on a tumor site-specific ‘wish-list’ containing hard constraints to be strictly obeyed and plan objectives with ascribed priorities. For BAO, a set of candidate beam orientations has to be defined as well (21). In this study, we used a published wish-list for prostate SBRT (14). This wish-list followed the above described planning approach, i.e. generate plans that strictly obey the constraints in **Table 1**, while obtaining adequate target coverage (highest priority), and maximally sparing the OARs (rectum, rectal mucosa, bladder, urethra, and femoral heads) and other healthy patient tissue, giving highest priority to high-dose rectum sparing, as suggested by QUANTEC (27). While adhering to constraints and imposed priorities, the ALARA principle for healthy tissues is inherently followed in automated plan generation with Erasmus-iCycle. For the optimizations with integrated BAO (VMAT+5 and 30-NCP), the non-coplanar beam selection search space consisted of 300 candidate beams, separated by about 10 degrees, and homogeneously distributed across the part of the sphere where collisions between the patient/couch and the gantry were avoided, as verified at the treatment unit (**Figure 1**).

All plans in this study were generated for an Elekta Synergy treatment machine equipped with a VersaHD collimator with a leaf width of 5 mm. 10 MV FF photon beams were used. Dose calculations in Monaco (version 5.10) were performed with a dose grid resolution of 3 mm. The total number of control points in all plans in this study was kept fixed at 300 for all investigated techniques (i.e. VMAT, VMAT+CS, VMAT+, and 30-NCP) to eliminate potential bias. IMRT was delivered with dynamic multi-leaf collimation with a maximum of 10 control points per involved beam, in line with our clinical practice. A fixed 5 degrees collimator angle was used for all arcs/beams, and the maximum dose rate was 600 MU/min. For generation of the VMAT+5 and VMAT+CS plans, VMAT and the non-coplanar IMRT beams were optimized simultaneously, both in Erasmus-iCycle and in Monaco. In this paper, ‘VMAT’ always refers to dual-arc VMAT with two full, 360° arcs.

### Workflow for Generation of the Non-Coplanar Beam Angle Class Solution (CS)

The final CS to supplement dual-arc VMAT in VMAT+CS treatments was developed in a stepwise approach, based on automatically generated plans:

- For each of the 20 study patients, the optimal VMAT+5 plan with individually optimized beam angles was generated.
- Based on an analysis of the angular distribution of the selected 20x5 non-coplanar beams, candidate class solutions, CS_i_, were defined as described in the Results section, all including a small number of frequently selected beam directions, and accounting for the left-right symmetry in the patients’ anatomies.
- For a subgroup of 6 randomly selected patients, VMAT+CS_i_ plans were generated for all CS_i_. These plans were then compared with corresponding VMAT plans, and the CS_i_ resulting in the most favorable plan quality increases relative to VMAT (focusing on rectum dose parameters) was selected as final CS.

### Dosimetric Comparisons of VMAT+CS Plan Parameters With VMAT, VMAT+5 and 30-NCP

Automatically generated VMAT+CS, VMAT, VMAT+5 and 30-NCP plans were compared for the 20 study patients. Prior to the analyses, all generated 80 plans were normalized to have identical PTV dose coverage (V_38Gy_=95%, as requested in clinical practice). Next, compliance with the clinically applied dose constraints (**Table 1**) was verified for all plans. Finally, plan parameter differences were analyzed. In line with the recently published RATING guidelines for planning studies (28), recommending the provision of a complete overview of dose differences, apart from the clinically evaluated plan parameters, we also evaluated and compared D_mean_ for both rectum and bladder, V_40GyEq_ and V_60GyEq_ (2 Gy/fx equivalent dose, i.e., V_22.9Gy_ and V_29.2Gy_, respectively) for rectum, as suggested by QUANTEC (27), as well as the dose bath, looking at patient volumes receiving > 30, > 20, > 10, > 5 and > 2 Gy. Paired two-sided Wilcoxon signed-rank tests were performed to assess clinical significance of observed differences (*p* < 0.05).

### Plan Deliverability, Treatment Time and MU for VMAT and VMAT+CS

For a subgroup of 5 patients with the largest plan quality gains achieved with VMAT+CS compared to VMAT, both VMAT and VMAT+CS plans were delivered at an Elekta Synergy linac (Elekta AB, Sweden) while irradiating a PTW 2D-Array seven29™ and Octavius™ phantom (PTW, Freiburg, Germany) at the corresponding couch angles. The measurements were compared to Monaco TPS predictions using a commercial QA software package (PTW VeriSoft version 6.2) with 5% cutoff, 3% global maximum dose and 1 mm distance to agreement (3%/1 mm) criteria, and 95% Gamma passing rate threshold. For delivery time comparisons, we separately measured *i)* beam-on-times, *ii)* gantry-travel-times (times to rotate the gantry from one fixed angle to another while the beam is off), and *iii)* couch-travel-times (times required to rotate the treatment couch in between beams, including time needed for entering the room). Additionally, the VMAT and VMAT+CS plans were compared regarding the total number of monitor units (MU).

## Results

### Establishment of the Final CS to Define VMAT+CS

**Figure 2:**
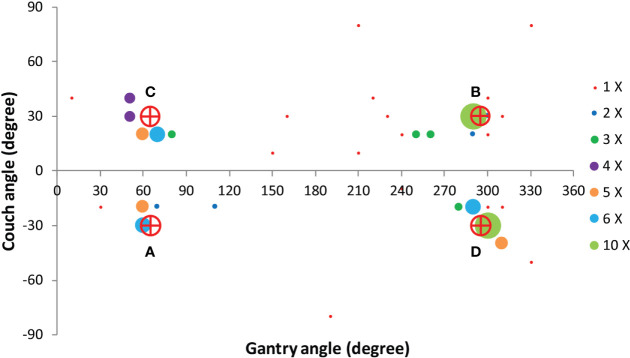
Angular distribution of the 100 non-coplanar IMRT beams in the VMAT+5 plans of the 20 study patients. Based on these results four principle beam directions A = (65°, -30°), B = (295°, 30°), C = (65°, 30°) and D = (295°, -30°) were derived to develop the final class solution for VMAT+CS.

**Figure 3:**
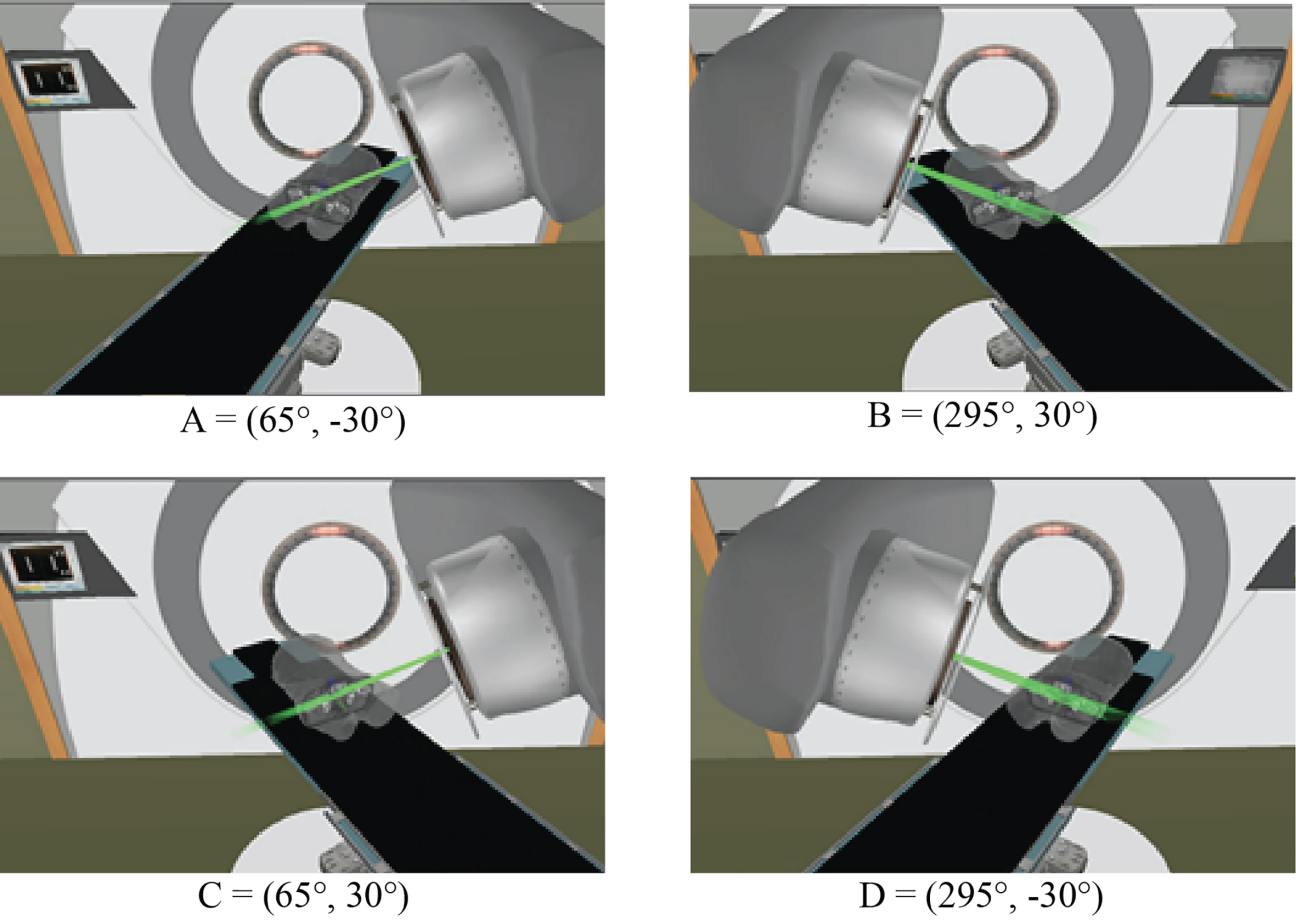
Four principle beam directions, characterized by (gantry angle, couch angle), from **Figure 2**, used to develop the final class solution for VMAT+CS. This final CS consisted of directions C and D.

**Figure 4:**
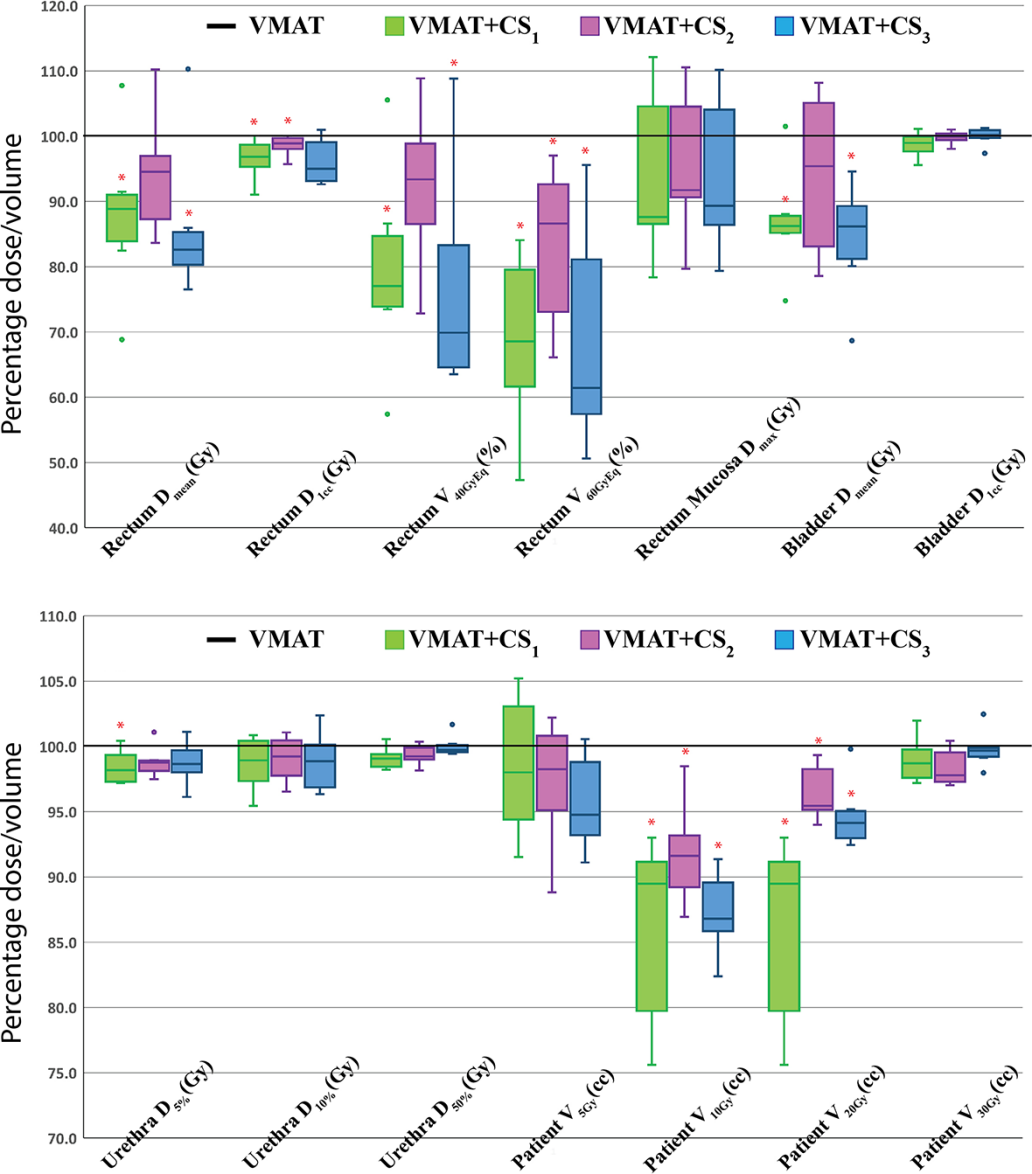
Healthy tissue dose parameters for VMAT+CS_1_, VMAT+CS_2_, and VMAT+CS_3_ plans relative to VMAT (VMAT always 100%) for the 6 randomly selected patients used to compare the class solutions. All plans were normalized to deliver the clinically required PTV coverage (V_38Gy_=95%). The central line of each box represents the median value, and its upper and lower edge the 25th and 75th percentiles, respectively. The whiskers extend to the minimum and maximum values, or to 1.5 times the inter-quartile range from the top/bottom of the box. Values outside this range are plotted individually as outliers (‘°’). *: difference with VMAT is statistically significant.

Based on the four clusters of frequently selected angles in the generated VMAT+5 plans (**Figure 2**), four principal directions (gantry angle, couch angle) for class solution definition were derived: A = (65°, -30°), B = (295°, 30°), C = (65°, 30°) and D = (295°, -30°). These are visually displayed in **Figure 3**. Based on these directions, three candidate CS_i_ were defined: CS_1_, containing all four directions, CS_2_ consisting of directions A and B, and CS_3_ with directions C and D. These CS are mutually compared for dosimetric performance in **Figure 4**. CS_2_ had clearly the highest median rectum parameter values and was therefore not selected for VMAT+CS. CS_3_ had lower median values for the rectum dose parameters than CS_1_. Differences between CS_3_ and CS_1_ in bladder D_mean_, bladder D_1cc_, urethra D_5%_, urethra D_10%_ and urethra D_50%_ were considered clinically irrelevant. The median patient V_5Gy_ and V_10Gy_ were better for CS_3_, while for V_20Gy_ and V_30Gy_ CS_1_ was better. Based on these observations and our clinical practice of giving high priority to low rectum doses, we selected CS_3_ to complement VMAT in VMAT+CS, and tested it for the whole patient group. In the remainder of this paper, VMAT+CS stands for VMAT+CS_3_.

### Comparisons of VMAT+CS Plan Parameters With VMAT, VMAT+5 and 30-NCP

**Figure 5:**
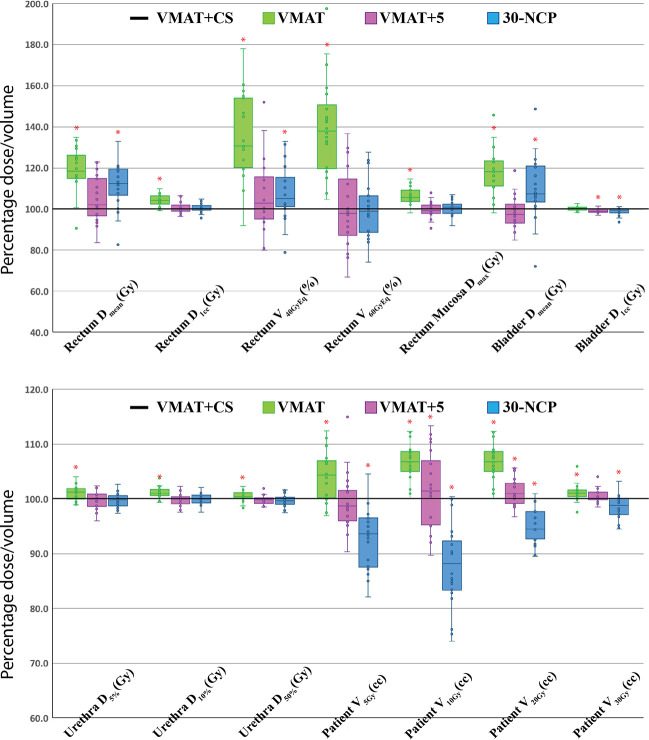
Healthy tissue dose parameters for VMAT, VMAT+5, and 30-NCP plans relative to VMAT+CS (VMAT+CS always 100%) for all study patients. All plans were normalized to deliver the clinically required PTV coverage (V_38Gy_=95%). The central line of each box represents the median value, and its upper and lower edge the 25th and 75th percentiles, respectively. The whiskers extend to the minimum and maximum values, or to 1.5 times the inter-quartile range from the top/bottom of the box. Hollow points are the discrete data points of the 20 study patients (‘°’). Positive values indicate lower doses for VMAT+CS. *: difference with VMAT+CS is statistically significant.

**Figure 6:**
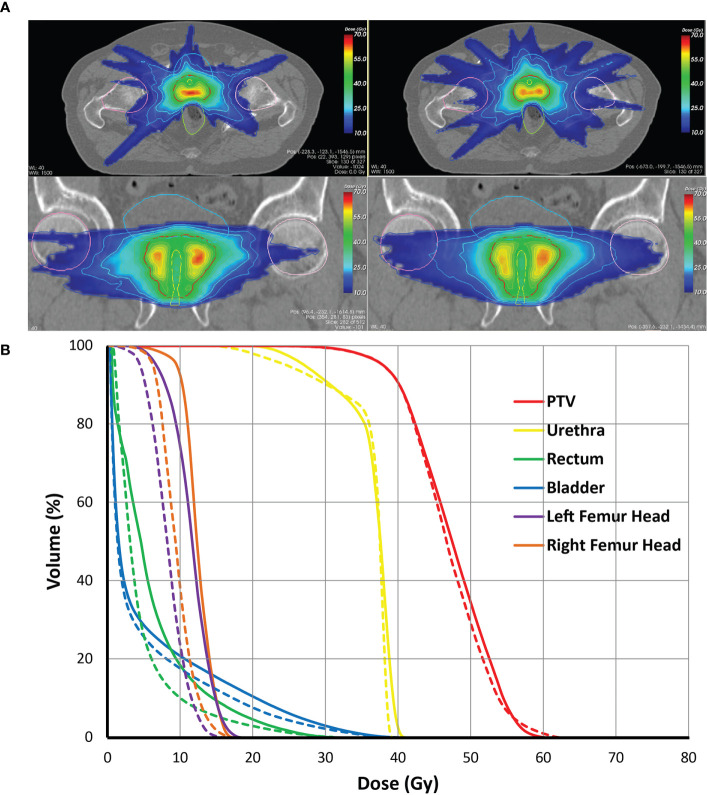
**(A)** Comparison of dose distributions for the VMAT+CS (left) and VMAT plans (right) for an exemplary patient on the axial, and coronal planes, **(B)** Population average dose-volume histograms for the VMAT (solid lines) and the VMAT+CS (dashed lines) plans of the entire patient cohort.

All 20x4 plans included in the analyses fulfilled the clinically applied dose constraints. In general, all plans with non-coplanar beam arrangements (VMAT+CS, VMAT+5, and 30-NCP) resulted in substantial reductions in doses in healthy tissues and dose bath compared to VMAT (**Figure 5** and **Table 2**). Differences between VMAT+CS and VMAT+5 were generally small and clinically negligible, while the former had less non-coplanar beams (yielding smaller overall treatment delivery time), and did not require individualized beam-angle-optimization (yielding reduced optimization time). Remarkably, also the differences between VMAT+CS and 30-NCP, the latter with much enhanced degrees of freedom in plan optimization, were relatively small. Actually, in rectum and bladder D_mean_ there was even a small advantage for VMAT+CS. For rectum D_1cc_, V_40GyEq_, V_60GyEq,_ bladder D_1cc_ and all urethra parameters, differences between VMAT+CS and 30-NCP were considered clinically irrelevant. There was a significant improvement in dose bath with 30-NCP, but clear disadvantages of this technique are the long optimization times (~100 hours per patient) and the very long delivery times (see below). **Figure 6** shows dose distribution of VMAT and VMAT+CS plans for an example patient, as well as the population average DVHs of the entire patient cohort.

**Table 2:** Comparison of dosimetric plan parameters of VMAT+CS with VMAT, VMAT+5, and 30-NCP plans for all study patients.

Structure	Parameter	VMAT+CS	VMAT – (VMAT+CS)		(VMAT+5) – (VMAT+CS)		30NCP – (VMAT+CS)	
		Mean ± SD [range]	Mean ± SD [range]	*p*-value	Mean ± SD [range]	*p*-value	Mean ± SD [range]	p-value
**PTV**	V_100%_ (%)	95.0 ± 0.0 [94.9, 95.1]	0.0 ± 0.1 [-0.1, 0.1]	1	0.0 ± 0.0 [-0.1, 0.0]	1	0.0 ± 0.0 [-0.1, 0.1]	1
	D_98%_ (Gy)	35.1 ± 0.7 [33.8, 36.3]	1.1 ± 1.5 [-1.0, 1.5]	**0.004**	-0.1 ± 1.6 [-2.8, 3.8]	0.3	0.6 ± 1.7 [-1.5, 4.3]	0.4
	CI	1.11 ± 0.04 [1.04, 1.20]	-1.6 ± 1.9 [-6.8, 2.5]	**0.001**	-0.5 ± 2.3 [-5.1, 3.4]	0.5	-1.5 ± 1.9 [-4.7, 2.0]	**0.01**
**Rectum**	D_mean_ (Gy)	5.4 ± 1.0 [3.7, 7.5]	19.4 ± 10.6 [-9.3, 35.0]	**<0.001**	4.6 ± 10.9 [-16.5, 23.0]	0.1	11.5 ± 11.2 [-17.4, 32.9]	**0.001**
	D_1cc_ (Gy)	27.6 ± 2.6 [23.7, 32.8]	4.2 ± 2.7 [-1.0, 9.8]	**<0.001**	0.5 ± 2.8 [-3.5, 6.5]	0.3	0.4 ± 2.1 [-4.3, 4.9]	0.6
	V_40GyEq_ = V_22.9Gy_ (%)	3.5 ± 1.3 [1.8, 6.1]	34.9 ± 20.3 [-8.1, 78.0]	**<0.001**	6.1 ± 18.5 [-20.1, 52.0]	0.2	7.6 ± 13.5 [-21.2, 32.8]	**0.03**
	V_60GyEq_ = V_29.2Gy_ (%)	1.1 ± 0.7 [0.2, 2.8]	39.7 ± 23.2 [4.6, 97.5]	**<0.001**	1.1 ± 19.9 [-33.2, 36.6]	0.8	-0.2 ± 13.6 [-25.8, 27.8]	0.5
**Rectum Mucosa**	D_max_ (Gy)	26.2 ± 2.6 [20.9, 31.6]	6.3 ± 3.9 [-1.9, 14.7]	**<0.001**	-0.3 ± 4.0 [-9.3, 8.0]	0.8	0.6 ± 3.6 [-8.2, 7.0]	0.6
**Bladder**	D_mean_ (Gy)	6.6 ± 1.3 [4.5, 8.8]	17.9 ± 11.0 [-1.8, 45.6]	**<0.001**	-1.5 ± 7.7 [-15.2, 18.6]	0.8	9.8 ± 15.7 [-28.0, 48.6]	**0.01**
	D_1cc_ (Gy)	36.7 ± 1.2 [34.1, 38.5]	0.3 ± 1.1 [-1.5, 2.7]	0.4	-1.0 ± 1.0 [-3.0, 1.3]	**0.04**	-1.1 ± 1.8 [-6.4, 1.1]	**0.02**
**Urethra**	D_5%_ (Gy)	40.1 ± 0.8 [38.6, 41.5]	1.1 ± 1.4 [-1.1, 4.0]	**0.003**	-0.4 ± 1.6 [-4.0, 2.4]	0.3	-0.2 ± 1.3 [-2.7, 2.7]	0.4
	D_10%_ (Gy)	39.6 ± 0.7 [38.4, 41.1]	1.1 ± 1.0 [-0.6, 3.8]	**<0.001**	-0.2 ± 1.1 [-2.4, 2.3]	0.6	-0.1 ± 1.0 [-2.4, 2.1]	0.6
	D_50%_ (Gy)	38.0 ± 0.5 [37, 38.9]	0.4 ± 0.9 [-1.7, 2.3]	**0.04**	-0.2 ± 0.8 [-1.5, 1.9]	0.9	-0.3 ± 1.1 [-2.5, 1.6]	0.3
**Left femur head**	D_max_ (Gy)	13.8 ± 1.6 [10.7, 17.9]	10.9 ± 8.4 [-8.8, 23.3]	**<0.001**	1.6 ± 8.3 [-15.5, 19.3]	0.1	-34.2 ± 15.6 [-65.4, -6.8]	**<0.001**
**Right femur head**	D_max_ (Gy)	14.0 ± 1.7 [10.7, 17.5]	7.4 ± 5.3 [-3.4, 16.9]	**<0.001**	-1.3 ± 4.7 [-9.2, 7.3]	0.7	-32.3 ± 13.9 [-50.0, -7.0]	**<0.001**
**Patient**	V_2Gy_ (cc)	5325 ± 1007 [4151, 7790]	9.3 ± 5.7 [-1.6, 20.5]	**<0.001**	18.6 ± 6.8 [6.3, 30.3]	**0.01**	32.9 ± 6.9 [14.7, 42.2]	**<0.001**
	V_5Gy_ (cc)	3444 ± 687 [2692, 5134]	4.1 ± 4.4 [-3.1, 12.4]	**0.001**	-0.6 ± 5.2 [-9.6, 14.9]	0. 6	-7.2 ± 5.5 [-17.9, 4.5]	**<0.001**
	V_10Gy_ (cc)	1332 ± 339 [924, 2022]	19.0 ± 7.3 [4.2, 30.4]	**<0.001**	1.4 ± 7.0 [-10.3, 13.3]	**0.01**	-12.2 ± 7.6 [-26.0, 0.3]	**<0.001**
	V_20Gy_ (cc)	317 ± 83 [213, 481]	6.7 ± 3.3 [0.1, 12.3]	**<0.001**	1.2 ± 2.4 [-3.2, 5.6]	**0.003**	-5.1 ± 3.2 [-10.5, 1.0]	**<0.001**
	V_30Gy_ (cc)	156 ± 44 [102, 246]	1.1 ± 1.6 [-2.5, 5.9]	**0.001**	0.5 ± 1.3 [-1.5, 4.1]	0.1	-1.6 ± 2.2 [-5.5, 3.2]	**0.01**

While the VMAT+CS column contains absolute values of the plan parameters, the VMAT, VMAT+5 and 30-NCP columns show percentage differences with respect to VMAT+CS. Positive differences hint at an advantage of VMAT+CS. Bold values denote statistically significance difference compared to VMAT+CS (i.e., p<0.05).

### Plan Deliverability, Treatment Time and MU for VMAT and VMAT+CS

**Figure 7:**
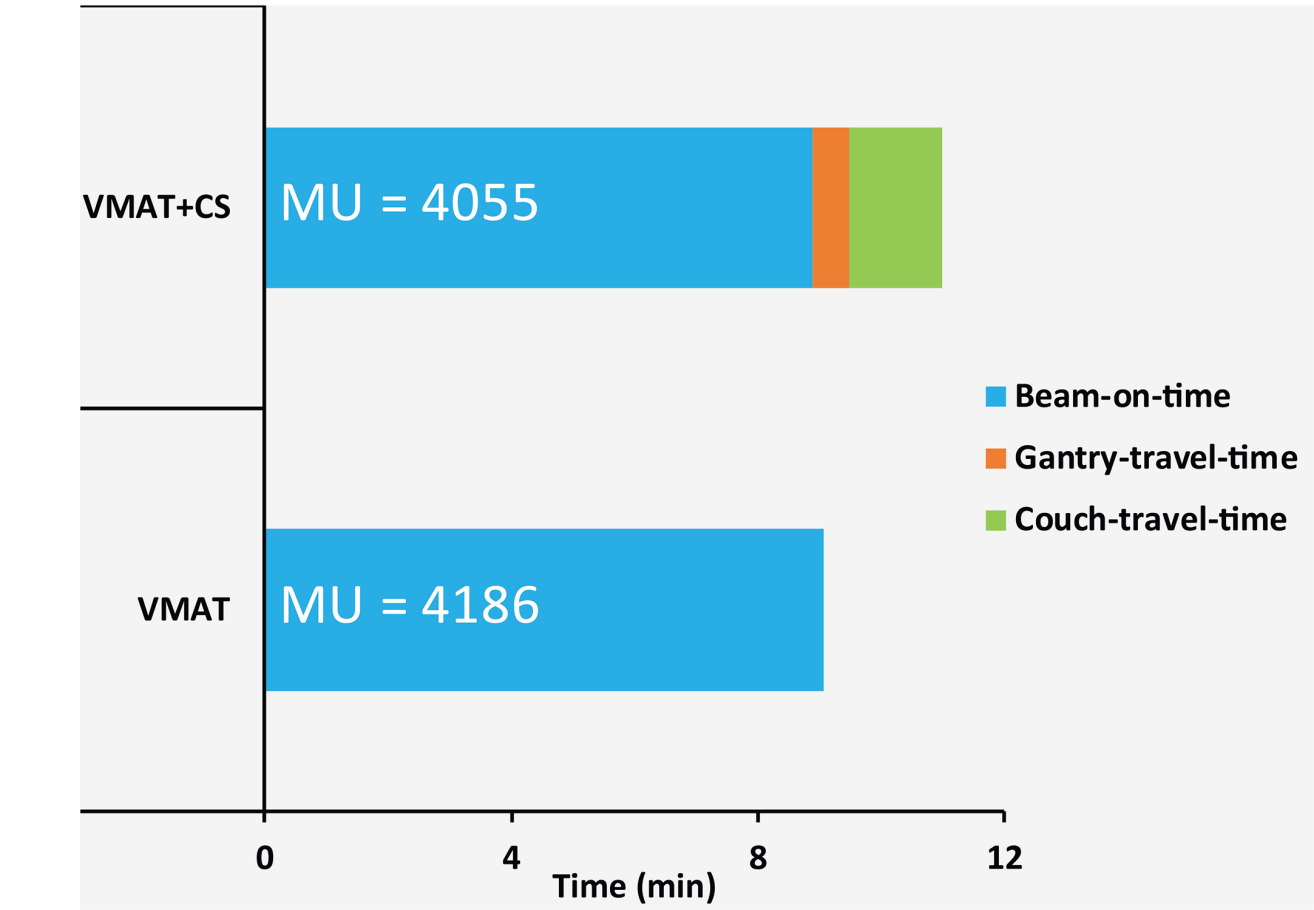
Average measured beam-on-times, gantry-travel-times and couch-travel-times for five VMAT and VMAT+CS plans. The numbers in the bars represent mean delivered MUs.

All delivered VMAT and VMAT+CS plans passed the QA tests (gamma passing rate >95%) with an average gamma passing rate of 98.3% ± 1.0% [97.5%, 100%] for VMAT plans and 98.3% ± 0.7% [97.5%, 99.4%] for VMAT+CS plans. Compared to VMAT, the average total delivery time of VMAT+CS plans increased from 9.1 ± 0.7 min to 11.0 ± 0.3 min (see **Figure 7** for details). VMAT+CS plans required 3% less MU (4055 ± 191 compared to 4186 ± 398), which was not significant (*p*=0.375). In VMAT+CS, on average, 15.7% ± 7.4% [4.9%, 33.3%] of the total MU was delivered by the two IMRT beams in the non-coplanar CS.

### Optimization Time Reduction

Using a class solution instead of individually optimized beam angle selection in VMAT+5 resulted in a substantial reduction in optimization time by a factor of 25. Optimization times for VMAT and VMAT+CS plans in Erasmus-iCycle took on average 50 and 60 min, respectively, while subsequent plan reconstruction in our Monaco TPS for either of the plans took on average 20 minutes.

## Discussion

In this study, we have developed and evaluated a novel treatment approach for prostate SBRT at a C-arm linac, consisting of dual-arc coplanar VMAT supplemented with a non-coplanar beam-angle class solution (CS) consisting of two IMRT beams (VMAT+CS). Initial aim of the study was to explore opportunities for enhancement of the plan quality for prostate SBRT, as obtained with VMAT, by adding non-coplanar beams. To keep the treatment delivery time short, no more than 5 non-coplanar beams were added. The study was inspired by the success for liver SBRT, where substantial plan quality enhancement compared to VMAT could be obtained by adding 1-5 non-coplanar beams with patient-specific, computer-optimized orientations (VMAT+) (19). For the prostate case studied in this paper, the distribution of selected non-coplanar orientations for VMAT+5 in the twenty study patients pointed at a possibility for the use of a CS, which was then successfully further explored. A fixed set of two non-coplanar orientations (CS) resulted for all patients in substantial plan quality enhancements compared to VMAT, while the increase in treatment time was very moderate (from 9.1 min to 11.0 min).

Remarkably, the quality of VMAT+CS plans was highly similar to that of VMAT+5 plans, the latter with more, and also patient-specific beam orientations. Most likely, because the pelvic anatomies of prostate cancer patients are highly similar, high quality plans could be generated for all patients with a fixed set of two non-coplanar beams supplementing VMAT. Interestingly, also the quality of plans with 30 non-coplanar beams with individually optimized orientations (30-NCP) was highly similar to that of VMAT+CS, while optimization and treatment times were largely enhanced. Apparently, adding only two well-selected orientations to the patient plans was enough for a substantial gain in plan quality. Adding more non-coplanar beams and patient-specific optimization of the orientations of the non-coplanar beams did not result in significantly better plans (except for some improvements in the low dose bath), especially when also considering the involved increases in optimization and delivery times.

In this paper, a CTV-PTV margin of 3 mm was used. Feasibility of margins depends on local set-up accuracies, e.g. determined by applied immobilization and image guidance procedures, available time for patient set-up, and training and ambition of RTTs. Last but not least, there is also the clinical trade-off between target coverage and OAR sparing. Recently, intra-fraction tracking with the Calypso system (Calypso Medical, Varian Medical System) has been introduced (29, 30) to maximally reduce the impact of intra-fraction prostate motion. Other investigators (31) have used an MR-linac for tracking of prostate tumors. Unfortunately, current MR-linac devices do not allow the use of non-coplanar beams.

Recently, Rossi et al. (14) showed a clear advantage for non-coplanar CyberKnife planning compared to coplanar VMAT for prostate SBRT, using the same study patients as in this paper. Also in that study, all plans were fully automatically generated, using the same autoplanning system and configuration (wish-list) as applied in this study. Interestingly, the quality of the VMAT+CS plans generated here is rather similar or slightly better than that of the previously generated CyberKnife plans (See **Table E1** for a comparison of dosimetric plan parameters). On the other hand, delivery times with the CyberKnife were much longer (45 min vs. 11 min in this study). There is a high similarity here with the above comparison between VMAT+CS and 30-NCP; apparently in prostate SBRT there is no need for using a large amount of non-coplanar beams to get a very high plan quality. Important to note is that CyberKnife allows tumor tracking to minimize the impact of intra-fraction prostate motion, allowing treatment with small (3 mm) margins (32). Without extra measures, such as monitoring and gating of treatment with a Calypso system (above), treatment at a regular C-arm linac could possibly result in lower effective tumor coverages for the same margins.

Currently, we have not yet an idea whether the CS developed for the planning protocol used in our center would also work for SBRT planning approaches in other centers. This is a topic of further research.

Addition of a few, well-selected non-coplanar beams to VMAT (the VMAT+ approach) has now turned out to be successful for both liver SBRT and prostate SBRT. New studies on other tumor sites are part of a future project. We are able to do this type of work due to availability of our in-house developed optimizer for fully automated and integrated multi-criterial beam angle selection and IMRT beam profile optimization. The success of our work on VMAT+ is an indication for the need of advanced algorithms for integrated beam angle and beam profile optimization in commercial treatment planning systems. In this context, one should keep in mind that with the VMAT+CS approach, BAO is not needed for new patients. However, development of the CS was only possible with the use of integrated optimization of beam angles and profiles.

To the best of our knowledge, this paper and our paper on liver SBRT (19) are the only papers that use autoplanning to systematically investigate the addition of non-coplanar IMRT beams to fast coplanar VMAT for enhancement of the plan quality achieved with VMAT, while keeping treatments fast. Several recent publications (33–37) investigated the use of non-coplanar arcs to enhance plan quality without prohibitively prolonged treatment times. Selection of the non-coplanar arcs was performed manually. Clark et al. (34) and Thomas et al. (35) showed that three non-coplanar arcs combined with one coplanar arc (at 0° couch angle) produced clinically equivalent conformity and dose spillage compared with GammaKnife for multiple cranial brain metastases while increasing the delivery efficiency due to its reduced treatment time. This class solution has been incorporated in the Eclipse treatment planning system as HyperArc and has further proven to improve delivery efficiency and reduce dose to normal brain tissue when compared to coplanar VMAT (38). More specific studies are needed to compare such approaches with the proposed VMAT+.

## Conclusions

Using an algorithm for fully automated, integrated multi-criterial beam profile and beam angle optimization, we have derived a two-beam non-coplanar class solution (CS) to supplement coplanar VMAT for prostate SBRT. Adding the CS beams resulted in substantial improvement in treatment plan quality with a minimal increase in treatment delivery time. Due to the use of a beam-angle CS, i.e. the same non-coplanar beam directions for all patients, time-consuming individualized beam angle optimization can be avoided in future use.

## Data Availability Statement

All relevant data are within the paper and its supplementary files. Access to raw-data underlying the findings in this paper will be made possible on request to corresponding author.

## Author Contributions

Conceptualization: AS, BH. Data curation: AS. Formal analysis: AS, LR, BH. Funding acquisition: BH. Investigation: AS, LR, MD, BH. Methodology: AS, LR, MD, BH. Project administration: AS, MD, BH. Resources: AS, LR. Software: AS, LR, SB. Supervision: MD, BH. Validation: AS, LR, SA, BH. Visualization: AS, LR, MD, BH. Writing original draft: AS. Writing review and editing: AS, LR, MD, SB, SA, BH. All authors contributed to the article and approved the submitted version.

## Funding

The authors declare that the department of Radiation Oncology of the Erasmus MC received funding from Elekta AB, Stockholm, Sweden and Accuray Inc, Sunnyvale, USA. The funder was not involved in the study design, collection, analysis, interpretation of data, the writing of this article or the decision to submit it for publication.

## Conflict of Interest

The authors declare that the research was conducted in the absence of any commercial or financial relationships that could be construed as a potential conflict of interest.
