# Supplementary material for: Complementing Prostate SBRT VMAT With a Two-Beam Non-Coplanar IMRT Class Solution to Enhance Rectum and Bladder Sparing With Minimum Increase in Treatment Time
Source: Front Oncol. 2021 Mar 19;11:620978. doi: 10.3389/fonc.2021.620978 (PMC8018286; doi:10.3389/fonc.2021.620978)
Supplement: Supplementary file 1 [file Table_1.docx]

***Table E1.*** *Comparison of dosimetric plan parameters of VMAT+CS plans in this study and CK plans in [14] for the same study patients. While the VMAT+CS column contains absolute values of the plan parameters, the CK-(VMAT+CS) column shows percentage difference with respect to VMAT+CS. For OARs, positive differences hint at an advantage of VMAT+CS.*

|  |  | VMAT+CS |  | CK – (VMAT+CS) | |
| --- | --- | --- | --- | --- | --- |
| Structure | **Parameter** | **Mean ± SD [range]** |  | **Mean ± SD [range]** | ***p*-value** |
| PTV | V_100%_ (%) | 95.0 ± 0.0 [94.9, 95.1] |  | 0.2 ± 0.1 [-0.1, 0.4] | **< 0.001** |
|  | D_98%_ (Gy) | 35.1 ± 0.7 [33.8, 36.3] |  | 2.7 ± 2.2 [-0.9, 6.9] | **0.001** |
|  | CI | 1.11 ± 0.04 [1.04, 1.20] |  | -7.1 ± 3.3 [-12.5, -0.9] | **< 0.001** |
| Rectum | D_mean_ (Gy) | 5.4 ± 1.0 [3.7, 7.5] |  | 16.9 ± 14.3 [-2.6, 44.1] | **0.02** |
|  | D_1cc_ (Gy) | 27.6 ± 2.6 [23.7, 32.8] |  | 0.3 ± 5.6 [-10.9, 10.7] | 1 |
|  | V_40GyEq_ = V_22.9Gy_ (%) | 3.5 ± 1.3 [1.8, 6.1] |  | 7.4 ± 27.6 [-31.9, 70.0] | 0.4 |
|  | V_60GyEq_ = V_29.2Gy_ (%) | 1.1 ± 0.7 [0.2, 2.8] |  | 2.1 ± 43.5 [-70.0, 84.7] | 1 |
| Rectum Mucosa | D_max_ (Gy) | 26.2 ± 2.6 [20.9, 31.6] |  | -1.2 ± 8.7 [-18.0, 15.8] | 0.7 |
| Bladder | D_mean_ (Gy) | 6.6 ± 1.3 [4.5, 8.8] |  | 45.2 ± 14.0 [24.3, 85.4] | **< 0.001** |
|  | D_1cc_ (Gy) | 36.7 ± 1.2 [34.1, 38.5] |  | 2.1 ± 2.8 [-2.0, 8.5] | 0.06 |
| Urethra | D_5%_ (Gy) | 40.1 ± 0.8 [38.6, 41.5] |  | 0.7 ± 2.6 [-3.7, 6.2] | 0.7 |
|  | D_10%_ (Gy) | 39.6 ± 0.7 [38.4, 41.1] |  | 0.7 ± 2.1 [-3.6, 5.5] | 0.4 |
|  | D_50%_ (Gy) | 38.0 ± 0.5 [37, 38.9] |  | 1.0 ± 1.2 [-1.9, 3.0] | **0.02** |
| Left femur head | D_max_ (Gy) | 13.8 ± 1.6 [10.7, 17.9] |  | 25.5 ± 15.0 [-6.3, 56.8] | **< 0.001** |
| Right femur head | D_max_ (Gy) | 14.0 ± 1.7 [10.7, 17.5] |  | 23.0 ± 14.5 [-5.0, 58.3] | **< 0.001** |
| Patient | V_2Gy_ (cc) | 5325 ± 1007 [4151, 7790] |  | 2.6 ± 20.3 [-41.8, 39.2] | 0.7 |
|  | V_5Gy_ (cc) | 3444 ± 687 [2692, 5134] |  | -7.3 ± 17.7 [-45.0, 18.8] | 0.4 |
|  | V_10Gy_ (cc) | 1332 ± 339 [924, 2022] |  | -13.0 ± 18.3 [-46.1, 18.4] | 0.06 |
|  | V_20Gy_ (cc) | 317 ± 83 [213, 481] |  | -4.5 ± 16.0 [-36.4, 19.3] | 0.7 |
|  | V_30Gy_ (cc) | 156 ± 44 [102, 246] |  | 0.0 ± 16.7 [-34.9, 24.6] | 1 |
